# The Influence of N-Acetylcysteine-Enriched Hydrogels on Wound Healing in a Murine Model of Type II Diabetes Mellitus

**DOI:** 10.3390/ijms25189986

**Published:** 2024-09-16

**Authors:** Albert Stachura, Marcin Sobczak, Karolina Kędra, Michał Kopka, Karolina Kopka, Paweł K. Włodarski

**Affiliations:** 1 Department of Methodology, Medical University of Warsaw, 1 Banacha Street, 02-091 Warsaw, Poland; 2 Doctoral School, Medical University of Warsaw, 1 Banacha Street, 02-091 Warsaw, Poland; 3 Department of Pharmaceutical Chemistry and Biomaterials, Faculty of Pharmacy, Medical University of Warsaw, 1 Banacha Street, 02-097 Warsaw, Poland; 4 Institute of Physical Chemistry, Polish Academy of Sciences, 44/52 Kasprzaka Street, 01-224 Warsaw, Poland

**Keywords:** wound healing, diabetes mellitus, acetylcysteine, biomedical hydrogels, hydrogel drug delivery systems, in vivo, db/db mice, leptin

## Abstract

Diabetes mellitus (DM) severely impairs skin wound healing capacity, yet few treatment options exist to enhance this process. N-acetylcysteine (NAC) is an antioxidant that improves cellular proliferation and enhances wound healing in healthy animals, yet its use in the context of type II DM has not been studied. The aim of our research was to investigate the effect of topically applied NAC-enriched hydrogels on wound healing in a leptin-deficient murine wound model. Four excisional wounds were created on the backs of 20 db/db mice and were subsequently treated with hydrogels containing NAC at concentrations of 5%, 10% and 20% or placebo (control). Healing was monitored for 28 days; photographs of the wounds were taken on every third day. Wound tissues were harvested on days 3, 7, 14 and 28 to undergo histological examinations. Wounds treated with 5% NAC showed improved wound closure speed accompanied by an increased dermal proliferation area on microscopic assessment compared with other groups. Higher concentrations of NAC failed to show a beneficial effect on wound healing. 5% NAC improved early stages of wound healing in a murine model of type II DM by increasing wound closure speed, likely mediated by improved dermal proliferation.

## 1. Introduction

N-acetylcysteine (NAC) is an antioxidant and a cysteine prodrug that potently replenishes intracellular glutathione levels [1]. It also regulates the expression of genes via inhibiting c-Jun N-terminal kinase, p38 MAP kinase or nuclear factor kappa B transcription factor [2]. NAC may prevent apoptosis and promote cell survival and directly reduces the activity of several proteins [2]. Though nonspecific, this substance has been widely used: as a mucolytic agent, an antidote to paracetamol intoxication, for doxorubicin cardiotoxicity, ischemia/reperfusion cardiac injury, acute respiratory distress syndrome and bronchitis [3]. Recently, it has been of interest to dermatologists and psychiatrists [4,5]. The dosages and administration routes vary greatly depending on the indication. High doses of up to 3000 mg/day orally or at a 20% concentration applied topically have been used in humans and were tolerated well [6,7]. 

The use of NAC has also been studied in the context of wound healing. This process comprises four consecutive phases: hemostasis, inflammation, proliferation and remodeling [8]. As NAC may reduce endothelial dysfunction and inflammation, as well as accelerate cellular proliferation [2], it has been considered a candidate intervention for wound healing enhancement. Topically applied 3% NAC promoted re-epithelialization in a rat model of burn wounds and, at the same concentration, improved angiogenesis and wound healing rate in an incisional wound model [9,10]. Tsai et al. showed the beneficial effect of NAC was dose-dependent and peaked at the maximum concentration of 3% [9]. Also, a single injection of 0.03% NAC decreased scar area and width in an incisional rat wound healing model [11]. This was associated with numerous changes in the gene expression patterns [12]. 

Diabetes mellitus (DM) severely impairs skin wound healing capacity. Factors contributing to dismal outcomes in patients treated for diabetic foot ulcer are poor angiogenesis and reduced blood flow, altered inflammatory response (often prolonged), proneness to infection, diminished amount of growth factors, accumulation of advanced glycosylation end products (AGEs), increasing oxidative stress, etc. [13]. NAC, with its mechanism of action, could theoretically alleviate some of these issues. It indeed improved the wound-breaking strength in an incisional alloxan-induced diabetic murine wound model following peritoneal NAC administration [14]. When NAC was administered systemically and/or topically to wounds in a rat, streptozotocin (STZ)-induced diabetic excisional wound model, wound areas decreased compared with the control group, which was associated with favorable histological outcomes [15]. Despite showing beneficial effects, these studies were limited by utilizing substance-induced type I DM models, which show relatively smaller wound healing impairment than leptin-deficient db/db type II DM model [16]. The latter model exhibits a wide range of healing dysregulations also seen in humans [17]. Moreover, the translatability of previous research is undermined by not addressing skin contraction—a major healing mechanism only present in rodents. A splinting model has been developed so that wounds heal through granulation and re-epithelialization, resembling the process occurring in patients with DM [18]. In our research, we took these methodological aspects into consideration. 

According to the Global Burden of Disease 2021 report, diabetes is the 10th leading cause of death and the 7th leading cause of disease burden worldwide [19]. The lifetime risk of developing a diabetic foot ulcer among people with diabetes mellitus ranges between 19% and 34% [20]. DM is also frequently associated with complicated wound healing [13]. Although surgical debridement remains the gold standard for the management of diabetic wounds [21], adjunct treatments are urgently needed to improve patients’ outcomes. Interventions such as growth factors, collagen scaffolds, chitosan gels and cold plasma therapy have been investigated to treat diabetic wounds. These potential therapies are, however, often costly or require specialized equipment. To date, only Becaplermin was approved for topical treatment of diabetic ulcers but was withdrawn soon after registration due to serious side effects [17]. Therefore, new easily accessible and effective alternatives should be investigated. As noted above, NAC was shown to improve wound healing in healthy animals and in some models of DM. It is also theoretically a more appealing molecule than modified growth factors as it is more easily accessible, cheaper and has a well-documented safety record [22]. This study aimed to broaden the understanding of NAC’s efficacy in improving diabetic wound healing using a hydrogel-based sustained-release formula.

Polymeric hydrogels are one of the most exciting medical materials for dermatology. One of the main advantages of hydrogels used in the topical treatment of skin diseases is their ease of application and significant minimization of side effects. Various combinations of natural, semi-synthetic and synthetic polymers are made into hydrogel formulations to use their potential as biomaterials. Hydrogels have numerous applications in the medical and pharmaceutical sectors [23,24,25]. This type of biomaterial can absorb large amounts of water or biological fluids while retaining integrity. Hydrogel drug delivery systems (DDSs) can change their properties in response to external or internal stimuli such as temperature, pH, ionic strength, etc. They are capable of releasing drugs in a prolonged and controlled manner [23,24,25,26]. Hydrogels are obtained from natural or synthetic polymers [24,27]. Their use helps maintain adequate wound moisture, which allows cell growth and migration. In a humid environment, keratinocytes can easily move around the wound’s surface, leading to its faster closure, and fibroblasts produce more collagen. A significant advantage of using hydrogels in wound treatment is their limited adhesion, which means they can be easily removed from the wound without causing further injury to the treated tissue [24,28]. Many hydrogel dressings have been developed for the treatment of difficult-to-heal wounds: poly(vinyl alcohol)(PVA)/β-glucan (β-1,6-branched-β-1,3-glucan) [29], dextran hydrogel [30], self-crosslink able dextran-isocyanatoethyl methacrylate-ethylamine hydrogel [31], hybrid dextran hydrogel with incorporated curcumin encapsulated (poly(lactide)-block-poly(ethylene glycol)) [32] and many others. 

The aim of this study was to discover the effect of topically applied NAC-enriched hydrogels on wound healing rate in a murine db/db excisional wound splinting model. Additionally, morphometric, histological and immunohistochemical staining analyses were performed to help elucidate the potential NAC mechanism of action.

## 2. Results

### 2.1. Hydrogel Characteristics 

**Figure 1:**
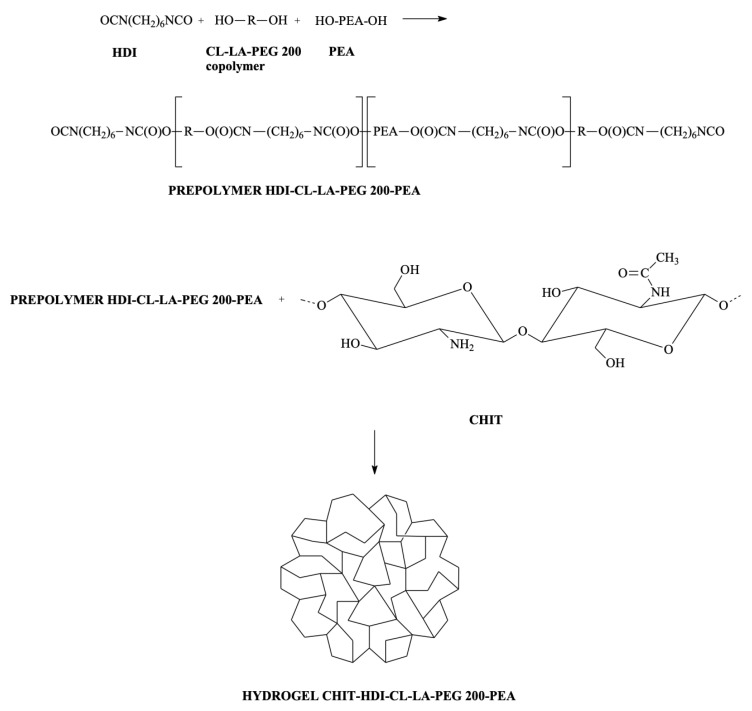
CHIT-HDI-CL-LA-PEG 200-PEA hydrogel preparation.

Hydrogels were obtained using a previously developed and appropriately modified three-step method with some modifications [33]. In the first step, є-caprolactone (CL), rac-lactide (LA) and poly(ethylene glycol) (PEG) (CL-LA-PEG 200) copolymer was synthesized. The prepolymer of 1,6-diisocyanatohexane (HDI), CL-LA-PEG 200 copolymer and poly(ethylene adipate) diol (PEA) were obtained in the second step. Next, chitosan (CHIT)-HDI-CL-LA-PEG 200-PEA hydrogel (HYDROGEL-MS1) was prepared in the final step (Figure 1).

**Figure 2:**
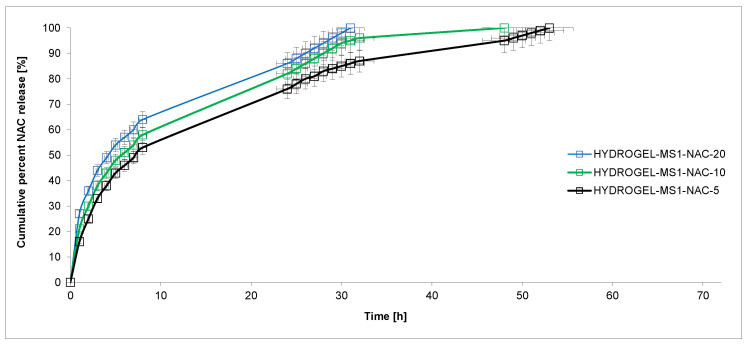
NAC release profiles from the obtained hydrogels (at pH 5.5) (each point represents the mean ± SD of three points).

The copolymer was synthesized by ring-opening polymerization (ROP) of CL, rac-LA and PEG 200 in the presence of immobilized lipase B from Candida antarctica (CA). The molas mass of the obtained CL-LA-PEG 200 copolymer was M_n_ = 2700 g/mol (D = 1.59). The copolymers’ molar content in the chain was 0.57 (CL): 0.38 (LA): 0.05 (PEG 200). The prepolymer was obtained in the polyaddition process of CL-LA-PEG 200 copolymer and PEA with HDI in an NCO/OH molar ratio of 0.9:0.1:2.1. Finally, the hydrogel was reacted with CHT in an NCO (prepolymer)/OH (or NH_2_) (CHT) ratio of 1.6:1. The dibutyltin dilaurate (DBDLSn) was used as a polyaddition catalyst. The swelling capacity of the obtained hydrogel was determined. The value of the coefficient of the mass swelling ratio (MSR) was 334%, 387%, 428% and 443% after 2 h, 4 h, 8 h and 24 h. NAC was loaded into hydrogels using the incorporation method. The mean weight of the devices developed was approximately 0.45 g, containing 5%, 10% or 20% of NAC (HYDROGEL-MS1-NAC-5, HYDROGEL-MS1-NAC-10 and HYDROGEL-MS1-NAC-20, respectively). In vitro studies of the release of NAC from obtained hydrogel materials were determined at pH 7.4 and 5.5 and at the temperature of 37 C for 72 h (Figure 2). The plot’s ordinate was calculated based on the cumulative amount of NAC released considering its initial amount in the hydrogels.

**Figure 3:**
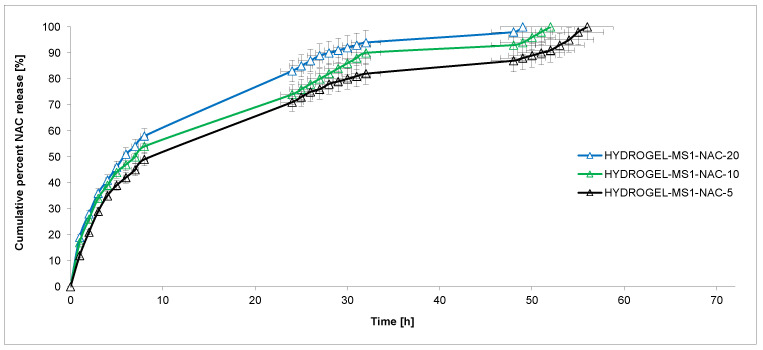
NAC release profiles from the obtained hydrogels (at pH 7.4) (each point represents the mean ± SD of three points).

The NAC release kinetics from obtained hydrogels were investigated (Figure 2 and Figure 3). The rate of NAC release at pH 7.4 decreased as follows: HYDROGEL-MS1-NAC-20 > HYDROGEL-MS1-NAC-10 > HYDROGEL-MS1-NAC-5. An identical trend was maintained for the experiments conducted at pH 5.5.

The results suggested that the NAC release rate increased as the pH of the solution decreased and the active substance contained in the hydrogel increased. For example, the percentage of the released NAC after 24 h incubation was about 83% from HYDROGEL-MS1-NAC-20 at pH 7.4 and 86% from HYDROGEL-MS1-NAC-20 at pH 5.5. After 24 h incubation, the NAC release was 83% from HYDROGEL-MS1-NAC-20, 74% from HYDROGEL-MS1-NAC-10 and 71% from HYDROGEL-MS1-NAC-5 at pH 7.4.

The data points obtained for the NAC release studies were subjected to zero- and first-order kinetics and the Korsmeyer–Peppas models to evaluate the kinetics and mechanism of NAC release from hydrogels (Table 1). According to the Korsmeyer–Peppas model, for the diffusion–degradation-controlled drug release system, the release exponent value *n* is in the range between 0.45 and 0.89 (anomalous, non-Fickian). In contrast, when n is close to 0.45, the diffusion (Fickian diffusion) predominates in the process, and, in the opposite case, *n* > 0.89, the model corresponds to the super case II transport [34].

The NAC release kinetic at pH 7.4 from HYDROGEL-MS1-NAC-5 and HYDROGEL-MS1-NAC-10 followed the near-zero-order model (R^2^ was 0.912 and 0.929, respectively). Furthermore, it was noted that NAC was released at pH 7.4 from HYDROGEL-MS1-NAC-20 with first-order kinetics (R^2^ was 0.989). In turn, when conducting the release process at pH 5.5, it was observed that for HYDROGEL-MS1-NAC-5, the NAC release was also close to the zero-order kinetics (R^2^ = 0.918). Furthermore, it was found that NAC was released from HYDROGEL-MS1-NAC-10 and HYDROGEL-MS1-NAC-20 with first-order kinetics (R^2^ was 0.954 and 0.925, respectively). The analysis of NAC release data using the Korsmeyer–Peppas model suggested that all hydrogels were governed rather by non-Fickian transport (*n* = 0.464–0.612). 

**Figure 4:**
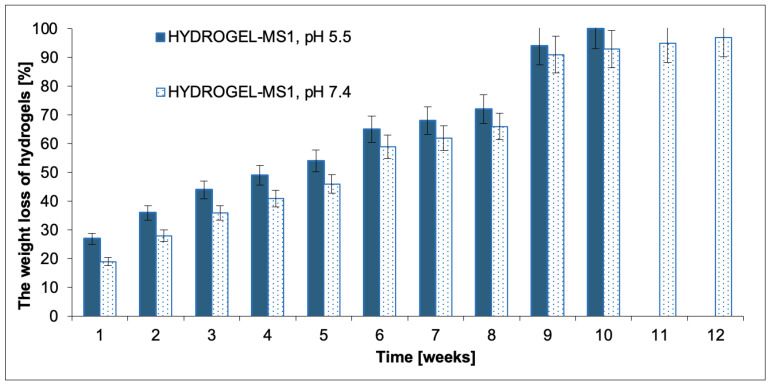
The weight loss of the obtained hydrogels over 12 weeks at pH 7.4 and 5.5 (each point represents the mean ± SD of three points).

The biodegradation of the blank hydrogels was also carried out. The hydrolytic degradation test of the resulting hydrogels was conducted under the same conditions as the NAC release experiments. The degradation process was characterized by plotting the weight loss (WL) of hydrogels against time. The results are shown in Figure 4. It was found that the biodegradation rate of hydrogels depended on the pH of the solution. Hydrogels biodegraded faster at pH 5.5 than at pH 7.4, which was found to be consistent with the NAC release experiment data.

The WL value for HYDROGEL-MS1 was 100% at pH 5.5 after 10 weeks of degradation. In turn, the WL value for HYDROGEL-MS1 was c.a. 100% at pH 7.4 after 12 weeks of degradation. Generally, the degradation process of obtained hydrogels was relatively slow and regular.

### 2.2. Wound Measurements

**Figure 5:**
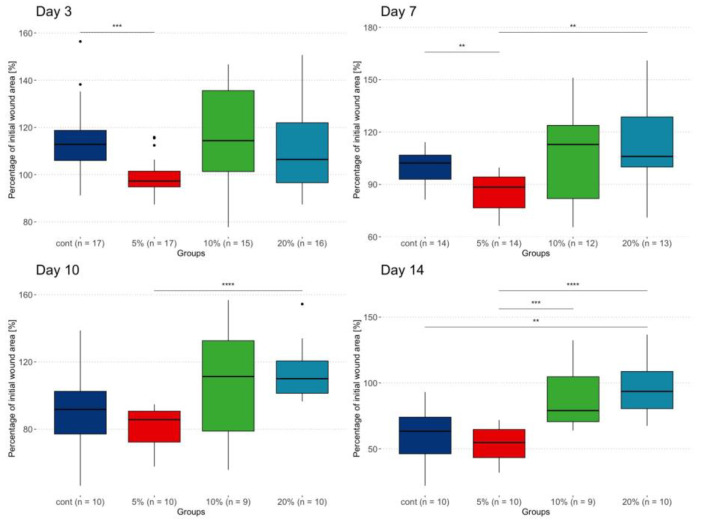
Wound area measurements on days 3–14. Displayed values represent the % of initial wound size. Statistically significant between-groups differences are marked with horizontal lines and significance levels (*p*-value): ** <= 0.01; *** <= 0.001; **** <= 0.0001.

During the first week, wounds treated with 5% NAC healed faster than those in the control group (Figure 5), and by day 7, their wound area was significantly smaller than those treated with 20% NAC. This difference remained significant until the end of week 2. On day 14, wounds treated with the highest NAC concentration also had bigger wound sizes than in the control group. At the same time, wounds treated with 10% NAC had larger areas than those treated with 5% NAC but not larger than in the control group. During weeks 3 and 4, no significant differences were observed between the groups. At that time, only five animals remained in each cohort (Table 2). 

### 2.3. Morphometry

Histomorphometric assessment included measurements of three key parameters: epidermal thickness, neoepidermal thickness and dermal proliferation area, i.e., the area of intense cellular proliferation adjacent to the wound (Table 3). Epidermal thickness was comparable between groups at most time points. The epithelium distal to the wounds gradually increased in thickness from day 3 to day 7 through day 14 and came back to near-baseline values on day 28. On day 7, epidermal thickness in the 10% NAC group was smaller than in the 5% NAC group (*p* = 0.006). The newly formed epithelium adjacent to the wounds was thicker than its distal counterpart. Similarly, it became thicker over time, reaching peak values on days 7 and 14. The new epidermis was thinner on day 7 in the group treated with 10% NAC compared with all other groups (*p* < 0.001). The dermal proliferation area was first measured on day 7, but its values became larger on day 14. DPA in the group treated with 5% NAC was more extensive than in the control group and 20% NAC group on day 7 (*p* < 0.006). 

### 2.4. IHC and Masson’s Trichrome Staining

Both iNOS and CD206 immunohistochemical staining resulted in high percentages of positively stained cells. No differences were detected between the studied groups. The signal from iNOS was least intense on day 7, whereas the CD206 signal was robust at the early stages of wound healing and decreased over time (Table 4). 

Masson’s trichrome allowed us to visualize collagen fiber orientation and density (Table 5 and Table 6). Throughout the study period, the degree of collagen alignment and density were comparable between groups. Only on day 28, the local directional variance was smaller in the 5% NAC group compared with the 10% NAC group (*p* = 0.007).

## 3. Discussion

We found that treatment with 5% NAC improved wound closure speed at early stages of healing—a finding that was linked with an increased dermal proliferation area on a microscopic level. We did not find significant differences in the expression of iNOS and CD206 in wound beds and did not capture significant differences in collagen fibers’ deposition and alignment between groups. Higher concentrations of NAC failed to enhance the wound healing process compared with control. In our project, we used hydrogels, which released NAC in a controlled and extended fashion, facilitating a prolonged action on the wounded tissue. Moreover, using the extended-release formula instead of instant-release administration allowed the delivery of the molecule less frequently, decreasing the amount of stress the animals were subjected to.

This is the first study investigating the effect of NAC on wound healing in an animal model of type II DM that we know of. Previously published research indicated a beneficial role of NAC in wound healing in the context of type I DM and healthy animals, both in excisional and incisional models. In our previous work, we showed that low-dose (0.03%) NAC reduced scar area and width at the early stages of wound healing in an incisional model in healthy rats [11]. This was associated with the persisting elevated expression of numerous genes involved in neoangiogenesis, proliferation and tissue remodeling [12]. Tsai et al. studied the effect of NAC on cell migration and proliferation in vitro and wound healing in a burn model in vivo. They found that NAC increased glutathione levels, cell viability and migration abilities in a dose-dependent manner. Additionally, topically applied 3% NAC improved re-epithelialization in vivo, and the effect was more pronounced in this group compared with lower concentrations [9]. Ozkaya et al. used NAC to treat excisional wounds in a streptozotocin-induced diabetic rat model [15]. They found that using either systemic or topical NAC resulted in improved epithelialization, lower fibrosis scores and reduced oxidative stress markers. Aktunc et al. applied NAC intraperitoneally to treat incisional wounds in two groups: healthy mice and mice with alloxan-induced diabetes [14]. They reported lower oxidative stress marker levels and increased wound-breaking strength in both groups following NAC administration. Our findings align with previous reports suggesting that NAC may improve re-epithelialization, which was reflected by an increased wound closure speed at the early stages of healing and by an increased dermal proliferation area, indicating improved proliferation and migration rates compared with other groups. Importantly, these effects were lost with NAC concentrations exceeding 5%. 

Despite encouraging preliminary in vivo reports, selecting an adequate dose and the NAC administration route is challenging. As shown above, NAC has not been repeatedly studied using uniform concentrations (literature reports range from 0.03% to 20%), routes of delivery (topical, intradermal injections or intraperitoneal), wound models (excisional, incisional or burn wound) and DM animal models (alloxan-induced, streptozotocin-induced or leptin-deficient). Though all mentioned studies reported beneficial results using NAC, it remains unclear which approach is optimal when it comes to molecule administration and indications. NAC has been used successfully at high concentrations in the clinical context to treat atopic dermatitis or acne vulgaris [6,35]. Acute treatment with high-dose NAC exhibits strong antioxidative and anti-inflammatory functions [36]—both of which are dysregulated in wound healing in DM [17]. In vitro NAC loses its pro-proliferative function in a dose-dependent manner [37]. We did not observe significant differences in iNOS and CD206 positively stained cells between groups at all time points. These are markers of M1 and M2 macrophages, respectively [38,39]. Faster attenuation of inflammation as measured with the density of these cells, as well as a quicker transition from predominant M1 to an M2 phenotype, has not been observed in NAC-treated groups. 

Given the pandemic of type II diabetes mellites and its global burden [40], as well as the scarcity of available topical treatments, future research should maximize its potential for clinical transferability. Excisional models using the splinting method allow the imitation of the natural process of granulation within human wounds and help overcome confounding wound contraction [41]. The diabetes mellitus model itself should also be carefully selected as it has been shown that wound healing in streptozotocin-induced DM (imitating type I DM) is not as impaired as in leptin-deficient mice [16]. Although the leptin-deficient model is not ideal, it has been used in many studies, and its biology is now well understood [17]. The authors of future studies should also consider choosing other animal models depending on available resources. Potential candidates for wound healing research include rabbit and pig models [42]. 

Our work has limitations. Firstly, murine wound healing models do not fully reflect the processes observed in humans due to the presence of the panniculus carnosus muscle, which plays a potent role in skin contraction. Therefore, excisional wounds heal primarily by this mechanism, not via the formation of granulation tissue, like in humans. We aimed to protect against this limitation by implementing a splinting model [41] and following a previously optimized wound creation protocol [18]. Secondly, large amounts of fat within the subcutaneous tissue made the histological specimens less concise when mounting onto the slides, creating artifacts and hindering analyses. Due to this limitation, we selected only those parameters that could have been measured reliably. Despite this step, the results of secondary analyses should be interpreted with caution. Thirdly, the sample size was sufficient to elucidate differences in the primary outcome measure between groups; however, secondary analyses may have been underpowered to detect small effects. Therefore, it cannot be stated that NAC does not influence the quantity of macrophages in the wound bed or does not have an impact on collagen alignment. These effects are possible, though a large effect is unlikely. Fourthly, splints mounted around the wounds could have fallen off throughout the study period and may have influenced consecutive assessments. We protected against unequal measurements of wound sizes by randomly allocating wounds to receive different treatments within each animal. 

## 4. Materials and Methods

### 4.1. Animals, Sample Size Calculation and Study Design

Animal care and handling were carried out under the UK’s Animals (Scientific Procedures) Act 1986 and associated guidelines under the EU Directive 2010/63/EU for animal experiments. The experiment was approved by the Second Local Ethics Committee in Warsaw (protocol code WAW2/029/2021). A total of 20 male 10-week db/db mice (strain BKS(D)-*Lepr^db^*/JorlRj, Janvier, Le Genest-Saint-Isle, France) were used in this experiment. Their baseline mean glucose serum concentration was 483 ± 191.9 mg/dL, and they weighted on average 50.2 ± 6.3 g. The number of animals needed for the experiment was based on a priori sample size calculation using G*Power 3.1 [43] (ANOVA: fixed effects, omnibus, one-way test) assuming a moderate effect size of 0.55, α error probability of 0.05, power of 95% and 4 groups. The effect size was estimated for the primary endpoint—wound healing rate. The final sample size was 64, but we increased the number of experimental units (wounds) to 80 (20 animals with 4 wounds each) due to a high risk of infection or early animal deaths. 

Four excisional wounds were created on the backs of each animal, and each wound within one animal was randomly assigned to one of the four groups: a control group receiving hydrogel without any active substance or one of three groups receiving hydrogels with different NAC concentrations: 5%, 10% or 20%. Group allocation was based on a computer random number generator. The allocation had been concealed before the experiment commenced by a person from outside of the research team, who tagged the hydrogel containers with one of four letters—A, B, C or D—representing one of the four interventions. Therefore, researchers were blinded to the intervention they were applying to each wound. Unmasking took place after the experiment had been finished. As each animal served as their own control, the risk of confounding was minimized. 

### 4.2. NAC Hydrogel Synthesis 

#### 4.2.1. Reagents

The following reagents were used for subsequent experiments: є-Caprolactone (2-Oxepanone, CL, 97%, Aldrich, Poznan, Poland), rac-lactide (3,6-dimethyl-1,4-dioxane-2,5-dione, rac-LA, 96%, Sigma-Aldrich, Poznan, Poland), 1,6-diisocyanatohexane (hexamethylene diisocyanate, HDI, 98%, Aldrich, Poznan, Poland), poly(ethylene glycol) 200 (PEG 200, M_n_ = 200 g/mol, pure, Sigma-Aldrich, Poznan, Poland), poly(ethylene adipate) diol (PEA) diol, M_n_ = 1000 g/mol, Sigma-Aldrich, Poznan, Poland), chitosan (CHIT, low molecular weight, 75% deacetylated, Sigma-Aldrich, Poznan, Poland), dibutyltin dilaurate (DBDLSn, >96%, Sigma-Aldrich, Poznan, Poland), immobilized lipase B from Candida antarctica (CA) (Sigma-Aldrich, Poznan, Poland), N-Acetyl-L-cysteine (NAC, ≥99.9%, Sigma-Aldrich, Poznan, Poland), dichloromethane (DCM, CH_2_Cl_2_, 99.8%, POCH, Gliwice, Poland), toluene anhydrous (Acros Organics, 99.8%, Extra Dry, Gdansk, Poland) and N,N-dimethylformamide (DMF, anhydrous, 99.8%, Sigma-Aldrich, Poznan, Poland). PEG 200 was used for copolymer synthesis, and HDI was were heated at 80 °C for 2 h in a vacuum to remove water residues. Phosphate buffer solution (pH 7.40 ± 0.05, 0.1 M, PBS, potassium dihydrogen phosphate/di-sodium hydrogen phosphate, 20 °C, Avantor Performance Materials, Gliwice, Poland) and potassium acetate buffer solution (100 mM, pH 5.5, 0.2 μM filtered, Avantor Performance Materials, Gliwice, Poland) were also used as received.

#### 4.2.2. Synthesis of є-Caprolactone, Rac-Lactide and Poly(ethylene glycol) Copolymers

The polymerization reactions were carried out according to our previously described method with some modifications [44,45,46]. Before the reaction, monomers (CL and rac-LA), PEG and CA were dried under a vacuum at room temperature for 2 h. Next, 0.05 mol CL and 0.05 mol rac-LA were placed in a three-neck flask equipped with a stirrer and thermometer (under argon atmosphere), and 20 mL of toluene was added. The mixture was stirred at 80 °C for 3 h. Next, an appropriate amount of PEG 200 and CA (500 mg) was added to the mixture. Stirring was continued at 80 °C for 72 h under an argon atmosphere. After this time, the enzyme was filtered off. Toluene was removed by evaporation under reduced pressure at room temperature. Next, the cooled product was dissolved in DCM and extracted with cold methanol and distilled water. 

Spectroscopy data of obtained copolymers are as follows: The ^1^H NMR: 1.40 ppm (-CO-CH_2_-CH_2_-CH_2_-CH_2_-CH_2_-O-), 1.48 ppm (-CO-CH(CH_3_)-O-), 1.64 ppm (-CO-CH_2_-CH_2_-CH_2_-CH_2_-CH_2_-O-), 2.30 ppm (-CO-CH_2_-CH_2_-CH_2_-CH_2_-CH_2_-O-) in -CO-Cap-Cap- sequences, 2.38 ppm (-CO-CH_2_-CH_2_-CH_2_-CH_2_-CH_2_-O-) in -CO-Lac-Cap- sequences, 3.63 ppm (-CH_2_-CH_2_-O-), 4.05 ppm (-CO-CH_2_-CH_2_-CH_2_-CH_2_-CH_2_-O-) in -CO-Cap-Cap- sequences, 4.13 ppm (-CO-CH_2_-CH_2_-CH_2_-CH_2_-CH_2_-O-) in -CO-Cap-Lac sequences, 4.21 ppm (-CH_2_-CH_2_-O-CH_2_-CH_2_-O-Cap-), 4.27 ppm (-CH_2_-CH_2_-O-CH_2_-CH_2_-O-Lac-), 5.04 ppm (-CO-CH(CH_3_)-O-); the ^13^C NMR: 17.02 ppm (-CO-CH(CH_3_)-O-), 20.49 ppm (-CO-CH(CH_3_)-OH) end groups, 24.62 ppm (-CO-CH_2_-CH_2_-CH_2_-CH_2_-CH_2_-O-), 25.57 ppm (-CO-CH_2_-CH_2_-CH_2_-CH_2_-CH_2_-O-), 28.39 ppm (-CO-CH_2_-CH_2_-CH_2_-CH_2_-CH_2_-O-), 32.36 ppm (-CO-CH_2_-CH_2_-CH_2_-CH_2_-CH_2_-OH) end groups, 34.16 ppm (-CO-CH_2_-CH_2_-CH_2_-CH_2_-CH_2_-O-), 62.57 ppm (-CO-CH_2_-CH_2_-CH_2_-CH_2_-CH_2_-OH) end groups, 63.49 ppm (-CH_2_-CH_2_-O-CH_2_-CH_2_-O-CO-), 64.18 ppm (-CO-CH_2_-CH_2_-CH_2_-CH_2_-CH_2_-O-), 66.77 ppm (-CO-CH(CH_3_)-OH) end groups, 69.21 ppm (-CO-CH(CH_3_)-O-) and (-CH_2_-CH_2_-O-CH_2_-CH_2_-O-CO-), 70.60 ppm (-CH_2_-CH_2_-O-), 170.90 ppm (-CO-CH(CH_3_)-O-) in lactyl units (L) in -Cap-L-Cap- sequences and 173.57 ppm (-CO-CH_2_-CH_2_-CH_2_-CH_2_-CH_2_-O-).

#### 4.2.3. Hydrogel Preparation and Characterization

Hydrogels were obtained using a previously developed and appropriately modified three-step method [33,47]. In the first step, the CL-LA-PEG 200 copolymer was synthesized. The prepolymer of DI and CL-LA-PEG 200 copolymer and PEA was obtained in the second step. Next, the CHIT-DI-CL-LA-PEG 200- PEA hydrogel was prepared. The prepolymers were obtained through a polyaddition reaction between HDI, CL-LA-PEG 200 copolymer and PEA in an NCO/OH molar ratio of 2.1: 0.9: 0.1, using 3 drops of 0.1 wt % DBDLSn solution in toluene as a catalyst. The reactions were performed at 80 °C for 3 h under an argon atmosphere to form an isocyanate-terminated prepolymer. Next, the CHIT dispersion into a glacial acetic acid/DMF mixture (30 mL) in a volume ratio of 50/50 was prepared. Next, the obtained prepolymer was added to the dispersion of CHIT. The reactions were carried out in an NCO (prepolymer)/OH (or NH_2_) (CHIT) molar ratio of 1.6:1 at 80 °C for 4 h under an argon atmosphere. The reaction mixture was then transferred to the distilled water. Precipitated products were separated by filtration and washed with DMF, methanol, and acetone. The final products were dried under a vacuum for one week. The mass swelling ratio (MSR) of obtained hydrogels was determined at 37 °C during 80 h of incubation in a buffer. Samples in triplicate were submerged in a buffer solution (20 mL) for a given time, and their weights were taken after removing the excessive surface water. The mass swelling ratio was calculated using the following formula:

MSR = ((W_2_ − W_1_)/W_1_)/100%

where W_1_ is the weight of the initial hydrogel and W_2_ is the weight of the swollen hydrogel.

To evaluate the percentage of degradation, the hydrogel samples were immersed in a buffer at 37 °C for 8 weeks; most importantly, the medium was replaced with a fresh buffer every week. At the end of the experiment, the samples were dried in a vacuum for 48 h. The degree of degradation of hydrogels (in triplicate) was determined by the weight loss (WL) of the samples according to the following equation:

WL = [(W_1_ − W_2_)/W_1_]/100%

where W_1_ is the weight of the dry sample before degradation and W_2_ is the weight of the dry sample after degradation.

#### 4.2.4. In Vitro Release Studies of N-Acetyl-L-Cysteine from Hydrogels

NAC was loaded to the hydrogel by physical mixing using the following procedure. A total of 5.0, 10.0 or 20% (m/m) of NAC in distilled water/Tween 80 mixture (2% (*w/v*) was added to three hydrogel samples (HYDROGEL-MS1-NAC-5, HYDROGEL-MS1-NAC-10 and HYDROGEL-MS1-NAC-20). The hydrogels were left sealed for 24 h. The mixtures were dried under vacuum at room temperature to obtain a NAC-loaded hydrogel film. The in vitro release of NAC from the hydrogels was performed in a buffer (pH 7.4 or 5.5) containing 2% (*w/v*) Tween 80 at 37 °C under stirring. Vials containing hydrogel films were filled with 5.0 mL of a buffer, sealed and left at 37 °C for 2 h. The solutions were then removed for further testing and replaced by fresh buffer. Subsequent samples were collected at selected intervals. NAC concentration in the in vitro samples was also determined by the UV-Vis spectrophotometric method (detected at a wavelength of 593 nm) [48]. 

The release data points were subjected to zero-order and first-order kinetics and Korsmeyer–Peppas models. Calculations were made based on formulas mentioned below [34]:

Zero-order: *F = kt*

$$\mathrm{First}-\mathrm{order}:\;\log\;F=\log\;F_{0}-\frac{kt}{2.303}$$

Korsmeyer–Peppas model: *F = kt^n^* (*F* < 0.6)

where *F* is the fraction of the drug released from the matrix after time *t*; *F_0_* is the initial amount of the drug, *k* is a model constant, and *n* is the drug release exponent in the Korsmeyer–Peppas model.

#### 4.2.5. Measurements

The structure of the obtained copolymers, M_n_, and the monomers conversion were evaluated using ^1^H and ^13^C NMR techniques. The spectra were recorded on an Agilent Technologies 400 MHz (Santa Clara, CA, USA) spectrometer. The synthesized copolymers M_n_ and *Đ* index values were determined using the GPC technique. The measurements were carried out on a Malvern Viscotek GPCMax TDA 305 (Malvern Panalytical, Malvern, UK) chromatograph equipped with a Jordi Gel DVB mixed bed column (Jordi Labs, Mansfield, MA, USA). The mobile phase flow (DCM) was set to 1.0 mL/min, and the column temperature was set to 30 °C. The system was calibrated using polystyrene standards. The amount of released NAC was determined by UV–Vis spectrophotometry (UV-1202 Shimadzu, Shimadzu, Kyoto, Japan) using a 1 cm quartz cell.

### 4.3. Surgical Procedure and NAC Administration 

All surgical procedures were performed using aseptic techniques. Male db/db mice aged 10 weeks (*n* = 20) were acclimatized to a 12 h light/dark cycle at 19 Celsius degrees, with water and high-energy-density food ad libitum. The animals were housed in a specific-pathogen-free room at the Central Laboratory of Experimental Animals, Medical University of Warsaw.

All steps of the surgical procedure were performed according to a previously published protocol with slight modifications [18]. Briefly, a few days before the experiment, 0.5 mm-thick silicone splinting rings with an 8 mm inner and 18 mm outer diameter were prepared. They were first washed with a detergent, rinsed with water, then incubated in sodium hypochlorite (20,000 p.p.m.) for 30 min, washed with sterile water and incubated in 70% ethanol for 30 min. They were air-dried on sterile gauze and kept in a sterile bottle before the surgical part commenced. On the day of surgery, all mice had the hair on their back cut, and removal cream was applied for 3–5 min and gently removed. Prior to surgery, grease was removed from the skin with a mild detergent.

The same anesthesia protocol was applied to all animals. Firstly, 2–3% isoflurane was used for induction in an anesthesia chamber, and later, anesthesia was maintained with 1–2% isoflurane mixed with normal air delivered via a mask. The skin was disinfected with octenidine dihydrochloride and phenoxyethanol, and four full-thickness 6 mm excisional wounds were created on the backs of each animal with a sterile single-use punch biopsy tool. This approach enables minimizing the number of animals used for the experiment and was previously validated in a leptin-deficient murine model [49]. An instant-bonding adhesive was used to mount the silicone splint so that the wound was centered within the splint. All splints were secured with three sutures of 6.0 nylon. Following the surgical procedure, all animals received an intramuscular injection with enrofloxacin 10 mg/kg and metamizole 30 mg/kg and a subcutaneous injection with buprenorphine 0.03 mg/kg. To avoid the drying of the cornea, Vidisic gel was applied topically on the eyeballs of all animals. All above-mentioned steps were performed by a surgeon unaware of the intervention allocation. 

**Figure 6:**
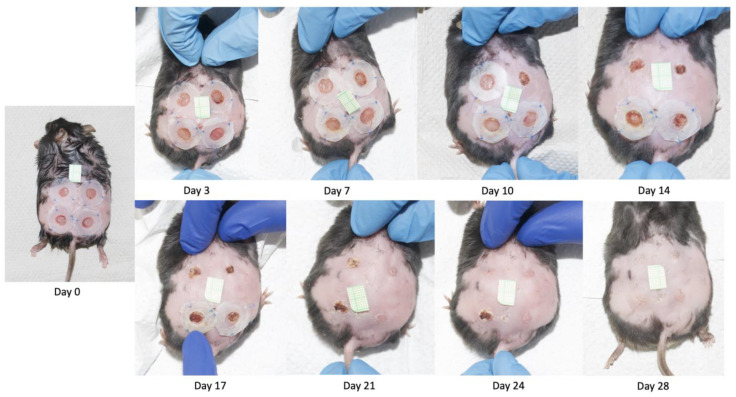
Examples of photographs taken to monitor the wound healing process throughout the experimental period.

Photographs of the wounds were taken with a digital camera (Figure 6), and hydrogels were applied to the wounds according to the pre-determined allocation based on letters representing study groups (A, B, C or D). The person applying hydrogels was blinded to the intervention used. The wounds and splints were covered with Tegaderm sterile transparent dressings, and mice were placed in separate cages with environmental enrichment to avoid biting wounds by cohabitants. 

NAC was topically applied on the wounds on the day of the surgery and subsequently on days 3, 6, 9 and 12 as the hydrogels were expected to gradually release active substances for approximately 2–3 days. All hydrogels were removed on day 14, and from that time, all wounds continued healing without any additional interventions. 

### 4.4. Tissue Harvesting and Histological Staining

**Figure 7:**
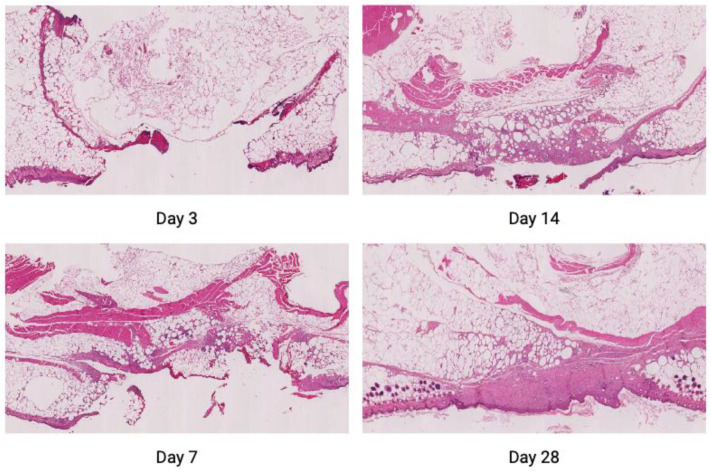
Examples of hematoxylin and eosin tissue staining images on days 3, 7, 14 and 28.

**Figure 8:**
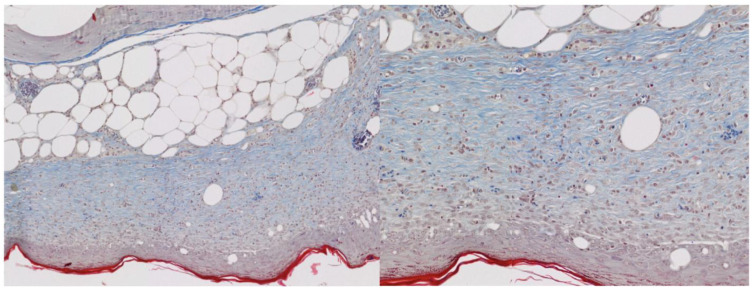
Examples of Masson’s trichrome staining images at two magnifications.

Five animals chosen at random were sacrificed on days 3, 7, 14 and 28. These days were selected to reflect different stages of wound healing. Typically, day 3 reflects the inflammatory phase, day 7 the proliferative phase and days 14 and 28 the remodeling phases; however, in diabetic wounds, these phases are usually delayed and may overlap. Wounds/scars were excised, divided into two equal parts and preserved for histologic and further analyses. One half was fixed in 10% formalin and embedded in paraffin using an automated tissue processor (ASP 6026, Leica, Buffalo Grove, IL, USA). Samples were sectioned into 3–5 µm slices, mounted on the histological slides and stained (1) with hematoxylin and eosin (Sigma-Aldrich, Saint Louis, MO, USA) using an automatic tissue stainer (Autostainer XL, Leica, Buffalo Grove, IL, USA); as shown in Figure 7, (2) with Masson’s trichrome (Sigma-Aldrich, Saint Louis, MO, USA) to visualize collagen fiber alignment (Figure 8); and using immunohistochemistry, described in a separate section. Manual staining protocols were followed according to the manufacturer’s instructions. All stained sections were scanned at 40x magnification using NanoZoomer XR C9600-12 (Hamamatsu, Iwata City, Japan).

Some wounds were excluded from analyses due to early animal death post-anesthesia (2 mice) or wound ulceration extending towards other wounds. Therefore, a total of 15 experimental units were excluded from subsequent analyses.

### 4.5. Macroscopic Wound Healing Assessment

Standardized photographic documentation of wounds was performed on the day of surgery and on days 3, 7, 10, 14, 17, 21, 24 and 28 following surgeries (Figure 6). The camera was placed on a stand 30 cm above the dorsal side of the animal. In each photo, there was a 1 cm-long millimeter scale placed on the skin. Photos were uploaded to ImageJ 1.48 v. software (National Institutes of Health, Bethesda, MD, USA) by a blinded researcher who measured the total wound areas. Results at each time point were presented as percentages of the baseline wound areas. 

### 4.6. Histomorphometry

Initially, we aimed to measure all critical wound parameters as described in our previous work [11]. However, as the subcutaneous tissue of leptin-deficient mice contains huge amounts of fat and the dermis is relatively thin, obtaining intact histological slides was challenging, particularly at the early stages of healing. To ascertain robust and reliable measurements and to reduce the chance of spurious findings, we decided to limit histomorphometric assessments to three parameters: thickness of the epidermis (measured thrice on each side of the wound), thickness of the newly formed epidermis (measured similarly) and dermal proliferation area excluding day 3. Measurements were performed as described in our previous work based on methods that had been introduced in works by Lemo et al. [50] and Schencke et al. [51]. 

### 4.7. Immunohistochemical Staining and Analysis

**Figure 9:**
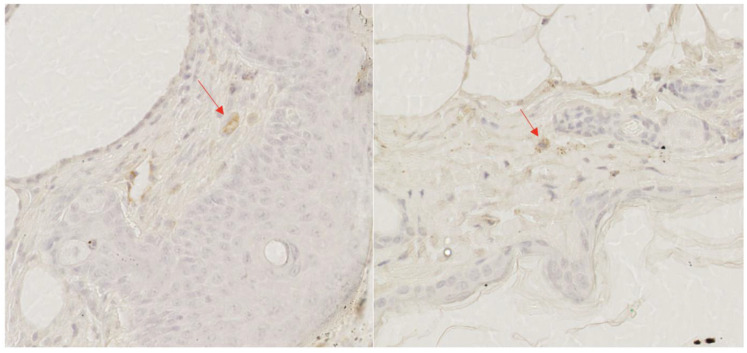
Examples of immunohistochemical staining images—anti-iNOS staining on the left and anti-CD206 staining on the right. Stained cells were marked with red arrows.

Following deparaffinization, immunohistochemistry staining was performed with REAL EnVision™ Detection System (DAKO, Agilent, Santa Clara, CA, USA, Code Number K5007). The following primary antibodies were used: anti-iNOS (Abcam ab115819) and anti-CD206 (Abcam ab64693) to visualize inflammatory cells, particularly different phenotypes of macrophages [38,39]. Two distinct staining methods were used as pro-inflammatory macrophages persist through day 10 following excisions in diabetic wounds and the macrophage polarization is skewed towards the M1 phenotype [17]. We aimed to assess whether NAC would improve the transition from the M1 to the M2 phenotype. Anti-iNOS antibodies were diluted 1:100 and incubated for 2 h, whereas anti-CD206 antibodies were diluted 1:20,000 and incubated for 15 min. The following reagents from DAKO were utilized during immunohistochemical staining: Wash Buffer (Code Number S3006); Peroxidase-Blocking Solution (Code Number S2023); Dako REAL TM Antibody Diluent (Code Number S2022); and Target Retrieval Solution, Citrate pH 6 (Code Number S2369). Staining procedures were carried out manually according to the manufacturer’s guidelines. All stained sections were scanned at 40× magnification with NanoZoomer XR C9600-12 (Hamamatsu, Iwata City, Japan) to obtain WSI (whole slide image) scans (Figure 9). WSIs underwent automated analysis in QuPath [52]. Uploaded files were manually checked for artifacts (stain traces, blood clots, folded tissue, etc.), and ROIs (regions of interest) were outlined. The process of nuclei identification was optimized, and the type of staining (intra- or extracellular) was selected. Results were presented as % of positively stained cells.

### 4.8. Statistical Analysis

For all variables, data distribution was verified using the Shapiro–Wilk test and double-checked if in doubt using QQ plots. For non-parametric data distribution, we used the Kruskal–Wallis test with the post hoc Wilcoxon test. For parametric data distribution, ANOVA was used with post hoc *t*-tests. All data were presented as means ± standard deviations for normally distributed data and as medians and IQR (interquartile ranges) for non-normally distributed data unless otherwise indicated. In the case of multiple comparisons, the Bonferroni correction was applied to avoid overstating significant differences. All statistical analyses were carried out in R version 4.2.3.

## 5. Conclusions

Topically applied hydrogel releasing 5% N-acetylcysteine improved the diabetic wound closure speed at early stages of healing, accompanied by an increased dermal proliferation area adjacent to the wound on histological assessment compared with the control hydrogel. Higher concentrations of NAC did not have a beneficial effect on the wound healing process in the leptin-deficient murine model of type 2 diabetes mellitus. We did not identify substantial changes in collagen fiber alignment or macrophage phenotype transition speed following NAC treatment. 

Future research should focus on optimizing the NAC concentration and the route of delivery in diabetic wound healing. In vivo studies should focus on exploring the mechanism of action of NAC, especially its potential to improve cellular proliferation. Experiments should closely reflect the wound healing conditions in humans. Therefore, if performed in murine models, they should apply splinting to counteract the robust contraction mechanism present in rodents.

## Figures and Tables

**Table 1:** Analysis data of NAC release from the obtained hydrogels.

**No.**	**Zero-Order Model**	**First-Order Model**	**Korsmeyer–Peppas Model**		**NAC Transport Mechanism**
	R^2^	R^2^	R^2^	n	
HYDROGEL-MS1-NAC-5 pH 7.4	0.912	0.905	0.951	0.561	non-Fickian transport
HYDROGEL-MS1-NAC-10 pH 7.4	0.929	0.903	0.988	0.612	non-Fickian transport
HYDROGEL-MS1-NAC-20 pH 7.4	0.835	0.989	0.998	0.536	non-Fickian transport
HYDROGEL-MS1-NAC-5 pH 5.5	0.918	0.909	0.956	0.540	non-Fickian transport
HYDROGEL-MS1-NAC-10 pH 5.5	0.861	0.954	0.996	0.486	non-Fickian transport
HYDROGEL-MS1-NAC-20 pH 5.5	0.873	0.925	0.997	0.464	non-Fickian transport

**Table 2:** Wound area measurements throughout the study period. Displayed values represent the % of initial wound size.

Timepoint		Control	5% NAC	10% NAC	20% NAC	*p*-Value *
Day 3	Median (IQR)	112.8 (106–118.7)	97.3 (94.8–101.5)	114.4 (101.3–135.6)	106.4 (96.6–121.9)	0.009
	Min–max	91.2–156.4	87.3–115.9	77.8–146.7	87.4–150.7	
Day 7	Median (IQR)	102.3 (92.9–106.8)	88.5 (76.6–94.3)	112.9 (81.9–123.8)	106 (100–128.7)	0.005
	Min–max	81.3–114.2	66.4–99.7	65.5–151.1	71–160.9	
Day 10	Median (IQR)	91.8 (77.1–102.5)	85.7 (72.3–90.7)	111.3 (78.9–132.6)	110 (101.3–120.6)	0.005
	Min–max	46.3–138.7	57.8–94.7	55.8–156.8	96.5–154.5	
Day 14	Median (IQR)	63.4 (46.2-74.1)	54.8 (43.3–64.7)	79 (70.6–104.7)	93.6 (80.5–108.7)	<0.001
	Min–max	22–93.2	31.9–71.9	63.9–132.4	67.5–136.7	
Day 17	Median (IQR)	52.6 (45.2–67.3)	41.4 (40.7–57.8)	63 (57.9–68.6)	83.5 (60.1–86.5)	0.11
	Min–max	19.7–74.8	24–68.9	50.9–70.8	56.5–100	
Day 21	Median (IQR)	0 (0–9.48)	37.9 (3.76–40.8)	14.3 (6.15–54.9)	57.9 (44.2–57.9)	0.33
	Min–max	0–65.3	0–48.3	3.3–61.2	2.5–59.9	
Day 24	Median (IQR)	0 (0–0)	0 (0–23.5)	0 (0–0)	40.5 (19.3–42)	0.42
	Min–max	0–51.7	0–49.2	0–46.5	0–48.5	
Day 28	Median (IQR)	0 (0–0)	0 (0–0)	0 (0–0)	0 (0–41.9)	0.89
	Min–max	0–56.4	0–59.4	0-44.5	0–57.5	

IQR—interquartile range, NAC—N-acetylcysteine; * Kruskal–Wallis tests.

**Table 3:** Histomorphometric measurement results in studied groups by time points.

Timepoint	Group	Epidermis Thickness [µm]	*p*-Value	Neoepidermis Thickness [µm]	*p*-Value	Dermal Proliferation Area [µm^2^]	*p*-Value
Day 3	Control	32.7 ± 14.2	0.07	53.9 ± 26.1	0.6	-	
	5% NAC	21.7 ± 7.75		57.5 ± 14.7		-	
	10% NAC	32.3 ± 19.4		53.8 ± 14		-	
	20% NAC	31 ± 11.9		61.3 ± 15.3		-	
Day 7	Control	31.8 ± 21.2	0.03	96.9 ± 22.1	<0.001	57,551 ± 29,408	0.003
	5% NAC	46 ± 33.8		104 ± 39.3		144,333 ± 28,204 ***	
	10% NAC	24.5 ± 9.45 *		60.6 ± 18.1 **		55,845 ± 33,352	
	20% NAC	40 ± 24		120 ± 20.3		50,684 ± 20,496	
Day 14	Control	45.8 ± 22.5	0.08	95.1 ± 33.3	0.4	248,247 ± 166,097	0.88 **
	5% NAC	49.3 ± 23.9		92.4 ± 44.9		379,831 ± 225,357	
	10% NAC	60.9 ± 21.9		96.3 ± 38.7		279,145 ± 99,181	
	20% NAC	51.7 ± 17.8		108 ± 39.2		416,778 ± 332,634	
Day 28	Control	24.4 ± 16.7	0.01	55.1 ± 20.6	0.2	246,033 ± 111,744	0.54 **
	5% NAC	27.7 ± 15.1		60 ± 16.8		441,084 ± 293,005	
	10% NAC	38 ± 21		69.7 ± 34.5		521,948 ± 468,316	
	20% NAC	26.2 ± 14		63.4 ± 27.6		307,195 ± 323,883	

All *p*-values are for between-groups comparison with ANOVA except DPA comparisons on days 14 and 28, where Kruskal–Wallis test was used due to non-normally distributed data. * *p* = 0.006 for comparison between 5% and 10% NAC. ** *p* < 0.001 for comparison between 10% NAC and all other groups. *** *p* < 0.006 for comparisons between 5% NAC and both control and 20% NAC. Remaining differences were insignificant after applying Bonferroni correction.

**Table 4:** Immunohistochemical staining analysis results. Values represent % of positively stained cells.

	iNOS					CD206				
Timepoint	Control	5% NAC	10% NAC	20% NAC	*p*-Value	Control	5% NAC	10% NAC	20% NAC	*p*-Value
Day 3	17.6 (13.6–29.9)	16.4 (15.8–40.8)	24.8 (22.2–27.3)	38 (34.5–46.6)	0.46	39.8 (33.9–47.6)	43.8 (24.9–59.8)	53 (50.8–56.2)	44.5 (40.6–52.5)	0.72
Day 7	19.6 (17.7–24.3)	15.3 (11–19.9)	13.7 (13.5–15.8)	10 (9.4–17.1)	0.57	32.4 (22.9–40.3)	30.2 (19.4–41.2)	30.3 (26.6–36.8)	36.1 (31.3–38.2)	0.98
Day 14	19.3 (15.2–22.9)	26.2 (19.4–41.5)	20.1 (16.5–23)	21.6 (19.9–22.9)	0.73	20.3 (16.3–39)	22.6 (19.3–22.8)	14.6 (11.7–27.7)	17.6 (10.6–26.3)	0.59
Day 28	23.8 (16.6–32.1)	29.4 (20.6–45.4)	18 (10.6–23.2)	24.9 (10.6–23.2)	0.39	19.7 (18–33.3)	10.2 (9.1–29.9)	14.8 (8.6–16.5)	18.3 (14.4–19.1)	0.3

**Table 5:** Masson’s trichrome staining analysis results in the proximal regions of the wounded tissue. For directional variance, values range from 0 to 1, corresponding to completely aligned and randomly aligned collagen fiber orientation, respectively. For collagen density, higher values indicate higher density.

Timepoint	Group	Directional Variance	*p*-Value	Local Directional Variance	*p*-Value	Collagen Density	*p*-Value
Day 3	Control	0.83 ± 0.05	0.21	0.65 ± 0.04	0.24	0.55 ± 0.12	0.17
	5% NAC	0.76 ± 0.06		0.55 ± 0.13		0.47 ± 0.25	
	10% NAC	0.85 ± 0.12		0.62 ± 0.1		0.45 ± 0.13	
	20% NAC	0.76 ± 0.09		0.61 ± 0.04		0.66 ± 0.08	
Day 7	Control	0.75 ± 0.12	0.15	0.61 ± 0.07	0.19	0.68 ± 0.15	0.25
	5% NAC	0.81 ± 0.05		0.66 ± 0.04		0.61 ± 0.14	
	10% NAC	0.79 ± 0.09		0.61 ± 0.07		0.58 ± 0.09	
	20% NAC	0.86 ± 0.06		0.67 ± 0.04		0.52 ± 0.14	
Day 14	Control	0.72 ± 0.07	0.27	0.59 ± 0.05	0.66	0.45 ± 0.13	0.54
	5% NAC	0.75 ± 0.08		0.58 ± 0.07		0.47 ± 0.18	
	10% NAC	0.79 ± 0.08		0.61 ± 0.06		0.49 ± 0.15	
	20% NAC	0.79 ± 0.09		0.61 ± 0.08		0.57 ± 0.22	
Day 28	Control	0.74 ± 0.13	0.07	0.59 ± 0.06	0.04	0.65 ± 0.13	0.007
	5% NAC	0.73 ± 0.11		0.55 ± 0.06		0.47 ± 0.09	
	10% NAC	0.83 ± 0.05		0.63 ± 0.05 *		0.66 ± 0.15	
	20% NAC	0.7 ± 0.09		0.57 ± 0.06		0.53 ± 0.11	

* *p* = 0.007 for comparison with 5% NAC; other pairwise comparisons non-significant after applying the Bonferroni correction.

**Table 6:** Masson’s trichrome staining analysis results in the distal regions of the wounded tissue. For directional variance, values range from 0 to 1, corresponding to completely aligned and randomly aligned collagen fiber orientation, respectively. For collagen density, higher values indicate higher density.

Timepoint	Group	Directional Variance	*p*-Value	Local Directional Variance	*p*-Value	Collagen Density	*p*-Value
Day 3	Control	0.77 ± 0.15	0.05	0.6 ± 0.1	0.18	0.53 ± 0.1	0.95
	5% NAC	0.68 ± 0.03		0.5 ± 0.12		0.48 ± 0.28	
	10% NAC	0.71 ± 0.12		0.53 ± 0.08		0.52 ± 0.09	
	20% NAC	0.87 ± 0.04		0.62 ± 0.05		0.53 ± 0.11	
Day 7	Control	0.72 ± 0.12	0.09	0.59 ± 0.08	0.04	0.59 ± 0.16	0.84
	5% NAC	0.79 ± 0.07		0.65 ± 0.03		0.58 ± 0.1	
	10% NAC	0.84 ± 0.03		0.63 ± 0.03		0.55 ± 0.04	
	20% NAC	0.82 ± 0.08		0.67 ± 0.03		0.54 ± 0.09	
Day 14	Control	0.67 ± 0.06	0.09	0.56 ± 0.05	0.69	0.51 ± 0.08	0.84
	5% NAC	0.77 ± 0.07		0.57 ± 0.07		0.49 ± 0.11	
	10% NAC	0.76 ± 0.11		0.6 ± 0.07		0.52 ± 0.13	
	20% NAC	0.76 ± 0.07		0.59 ± 0.07		0.55 ± 0.19	
Day 28	Control	0.79 ± 0.06	0.66	0.62 ± 0.05	0.23	0.65 ± 0.09	0.72
	5% NAC	0.73 ± 0.11		0.56 ± 0.07		0.64 ± 0.1	
	10% NAC	0.75 ± 0.14		0.56 ± 0.08		0.6 ± 0.14	
	20% NAC	0.77 ± 0.05		0.6 ± 0.05		0.67 ± 0.09	

## Data Availability

The data that support the findings of this study are available from the corresponding author, P.K.W., upon reasonable request.
